# One-year incidence of sexual harassment and the contribution to poor mental health in the adult general population

**DOI:** 10.1093/eurpub/ckab225

**Published:** 2022-01-29

**Authors:** Per-Olof Östergren, Catarina Canivet, Anette Agardh

**Affiliations:** Division of Social Medicine and Global Health, Department of Clinical Sciences Malmö, Lund University, SE-205 02 Malmö, Sweden; Division of Social Medicine and Global Health, Department of Clinical Sciences Malmö, Lund University, SE-205 02 Malmö, Sweden; Division of Social Medicine and Global Health, Department of Clinical Sciences Malmö, Lund University, SE-205 02 Malmö, Sweden

## Abstract

**Background:**

Sexual harassment (SH) has been highlighted as an important determinant for mental health. The aims of this study were to describe SH in terms of cumulative 1-year incidence, exposed groups, types of perpetrators and settings, and to measure the association between SH and poor mental well-being.

**Methods:**

Data from two waves of the Scania Public Health Cohort Study, comprising 7759 randomly recruited individuals above 18 years. Exposure to SH was measured by an instrument that also recorded place of exposure and type of perpetrator. Poor mental well-being was assessed by General Health Questionnaire (GHQ)-12, 36-Item Short Form Health Survey questionnaire (SF-36) (mental health module) and self-reported use of prescribed psychotropic medication. Logistic regression was used for multivariate analyses.

**Results:**

The cumulative 1-year incidence of SH was six times greater among women, the highest figure (17.5%) was noted for women 18–34 years of age. No difference was noted regarding educational level or country of origin. Public places, including public transportation, and unknown offenders were the most frequently reported setting of SH for both genders. Exposure to SH was associated with a doubled risk for low mental well-being, according to all three outcome definitions, and after adjustment for mental health 6 years earlier. Female gender was related to greater vulnerability to SH exposure concerning GHQ-12 and SF-36. The Population Attributable Fraction for poor mental health was calculated to 13% for women 34 years and younger.

**Conclusion:**

The results of this study make SH an important issue for gender policy as well as for public health policy and intervention.

## Introduction

A recent European study estimated that 45–55% of women in the EU have been subjected to sexual harassment (SH) since the age of 15 years, and 13–21% during the last 12 months.^1^ SH in workplace and educational settings has been extensively studied and shows no clear signs of decreasing prevalence rates.^2^^,^^3^ The issue of street and public transport SH is lately gaining enhanced research interest.^4–7^ Since there are many studies reporting links between SH and poor mental health outcomes,^8–12^ as well as to decreased job satisfaction and long-term sickness absence,^13–15^ exposure to SH stands out as a major public health issue. However, most of the previous studies have focussed on young individuals or on the workplace or educational institution, or only on women. Hence, studies addressing the full adult population seem comparatively scarce.

A national general population survey, performed in 2017 by The Public Health Agency of Sweden, showed that 42% of the women and 9% of the men had been exposed to SH during their lifetime.^16^ Since then, the Swedish manifestations of the #MeToo movement,^17^ signed in by over 75 000 individuals, have demonstrated the pervasiveness of SH in various sectors of the Swedish society.^18^^,^^19^

However, data on where the SH takes place are scarce, except for data on SH reported by The Swedish Work Environment Authority. In 2015, 19% of women and 6% of men 16–29 years old affirmed SH at the workplace during the past year.

Detailed information concerning SH and its impact on health and productivity can not only fill important knowledge gaps, but is also vital for identifying groups that potentially benefit from targeted interventions.

### Aim of the study

The aim of the study was to determine the cumulative 1-year incidence of SH among women and men in different ages and socioeconomic groups in Sweden and the association between SH and mental well-being, on the individual level as well as on the population level (population attributable fraction).

## Methods

### Study population

The Scania Public Health Cohort (SPHC) was established in 1999/2000 when a questionnaire, containing more than 120 questions about sociodemographic background factors, living and working conditions, health behaviours and self-rated health, was sent out by regular mail to 23 437 randomly selected men and women, 18–80 years old.^20^ The response rate was 58% (*N* = 13 589). These individuals were invited to follow-up, with similar questionnaires, in 2005 and 2010; the response rates were 81 and 75%, respectively. At the follow-up in 2010, the cohort was complemented with an invitation to 4030 individuals in the age range 18–27 years. Comparisons with official registry information from corresponding age cohorts showed a low risk for selective bias in the SPHC cohort,^21^ and extended reweighting analyses confirmed these findings.^22^ In 2016, a new follow-up was performed, adding 6596 individuals aged 18–27 and 28–34 years. The total response rate in 2016 was 45%. Information for the present study is derived from the 2016 assessment (*N* = 7759), while in the multivariate analyses, data from the 2010 inquiry is also used, limiting the number of participants to 5886.

### Background variables

Information from the national population register was used for dichotomization into women and men. Country of origin was recorded as ‘born in Sweden’ and ‘not born in Sweden’. Education level at baseline was determined by the self-reported total years of formal education and dichotomized as ‘≤ 12 years’ and ‘≥ 13 years’, which according to the Swedish educational system results in one group with maximum secondary education and another group with more than secondary education.

### Outcome measure—poor mental health

Mental health was assessed by three measures, first the 12-item version of the General Health Questionnaire (GHQ)-12. We used the 0-0-1-1 scoring method (range 0–12) with poor mental health defined as a score of 2 or higher^23^^,^^24^ (here labelled ‘GHQ-case’). We further included the Mental Health subscale of the 36-Item Short Form Health Survey questionnaire (SF-36).^25^ We used the threshold of ≤56, which in a recent study detected either depression or anxiety with a sensitivity of 87.9%, specificity of 76.9% and accuracy of 80.6%,^26^ and this was labelled ‘SF36 depression/anxiety case’. Finally, participants’ affirmative answer to a survey question about use of medication against general worry, anxiety or sleeping problems during the past 3 months was used as a third indicator of poor mental health; labelled ‘Self-reported use of psychotropic medication past 3 months’. These three outcome variables were statistically significantly correlated (*P* < 0.0001) with the following Pearson values: between GHQ-case in 2016 and SF depression/anxiety: 0.56, between GHQ-case in 2016 and use of psychotropic medication: 0.18, and between SF depression/anxiety and use of psychotropic medication: 0.25.

### Exposure—SH

SH was assessed with the following question (yes/no): *‘Have you, during the past 12 months, been subjected to sexual harassment? (Sexual harassment refers to all types of unwanted sexual advances directed towards oneself.)’* Participants were also asked to specify where the instance/instances of harassment took place and the category of the offender/s. Several alternatives could be chosen for both questions. The answer alternatives for location were ‘at work’, ‘at the university’, and ‘at other school/training facility’, ‘in my home’, ‘in somebody else’s home’, ‘in a public place/entertainment venue/on a train/bus/underground’ and ‘other place’. The answer alternatives for type of offender were ‘regular partner’ (for more than a month) and ‘temporary partner’, ‘family member or close relative’, ‘person working at my workplace’, ‘person that I met through work (customer, patient, client, care recipient, student, etc.)’, ‘unknown person’ and ‘other person’.

The study was approved by the Regional Ethical Review Board at Lund University, Sweden (1999-99; 2005-471, 2010-392, 2015-471, and 2016/622).

### Statistical methods

The relationships between background factors, SH and poor mental health are presented as percentages, with p for chi square values, and age-adjusted odds ratios, with 95% confidence intervals. The tests for effect modification were performed with simple dummy variables. The synergy indexes were calculated as proposed by Rothman,^27^ according to which a synergy index > 1 indicates a synergistic effect and a synergy index < 1 an antagonistic effect. In the multivariate analysis, SH is tested against GHQ status in 2016 with the stepwise addition of age group, sex and GHQ status in 2010.

The population attributable fractions (PAF) (i.e. the proportion of disease cases over a specified time that would be prevented following elimination of the exposures, assuming the exposures are causal)^28^ for SH in relation to poor mental health (defined as GHQ-case) were calculated, using the formula: PAF = pd × (OR − 1)/OR,^28^ where pd is the proportion of cases exposed to SH, and OR is the OR for poor mental health. This was done for women in two age groups, but not for men, since the number of exposed men were too few.

A standard statistical analysis programme was used (SPSS version 25.0).

## Results

Exposure to SH during the past year was affirmed by 3.5% of the entire population (table 1). It was six times more frequent among women compared with men (OR: 6.2; 95% CI: 4.2–9.1) and as expected, much more frequent among younger persons. Among women aged 34 years and below, the prevalence for having been exposed to SH during the past 12 months was 17% and the risk was nine times higher, compared with women above this age. Neither educational level nor country of birth seemed to affect the risk when adjusted for age.

**Table 1:** Associations between background factors and exposure to sexual harassment during the past 12 months

ns (missing)		Not exposed		Exposed		*P* for chi-sq	OR	95% CI
		ns	%	ns	%			
Age groups (176)	18–24	390	83.2	79	16.8		19.5	13.3–28.4
	25–34	774	90.1	85	9.9		10.6	7.3–15.2
	35–44	623	96.1	25	3.9		3.9	2.4–6.3
	45–54	1112	97.4	30	2.6		2.6	1.6–4.1
	55 and older	4419	99.0	46	1.0	<0.0001	1	
	Total	7318	96.5	265	3.5			
Gender (176)	Men	3237	99.1	30	0.9		1	
	Women	4081	94.6	235	5.4	<0.0001	6.2	4.2–9.1
Age and gender groups (176)	Male ≤ 34 years	459	97.0	14	3.0		5.3	2.6–10.9
	Male ≥ 35 years	2778	99.4	16	0.6		1	
	Female ≤ 34 years	705	82.5	150	17.5		36.9	21.9–62.3
	Female ≥ 35 years	3376	97.5	85	2.5	<0.0001	4.4	2.6–7.5
Educational level^a^ (355)	≤ 12 years	3420	96.7	110	3.3		1.1	0.9–1.4
	≥ 13 years	3904	96.3	150	3.7	0.34	1	
Born in Sweden^a^ (231)	Yes	6654	96.6	236	3.4		1	
	No	611	95.8	27	4.2	0.31	1.0	0.7–1.6

**Notes:** Scania Public Health Cohort 2016, *N* = 7759. Odds ratios (OR) and 95% confidence intervals (CI) for exposure to SH are presented.

a ORs are age-adjusted.

Regarding the reported place for exposure to SH (table 2), public places (including public transportation) was the most frequently reported location both for men and women, 40.0% and 53.2%, respectively, with the younger age group, i.e. those 34 years and younger, reporting the highest frequencies, both among men and women. Men tended to report that they had experienced SH comparatively more often in their own or someone else’s home or in an educational setting (university or school or training situation), while women had encountered SH comparatively more often at a workplace. Another gender difference was that women in the older age group (35 years and older) seemed relatively more often exposed to SH at their workplace (36.5%) than men in the same age group (12.5%, or 2 out of 16 exposed individuals). None of these differences were statistically significant.

**Table 2:** Location where sexual harassment took place, and offender, by age and gender groups of the 265 persons (30 men and 235 women) who affirmed having been exposed to any such action during the past 12 months. Several alternatives could be chosen

	Men ≤ 34 years (*N* = 14)		Men ≥ 35 years (*N* = 16)		Women ≤ 34 years (*N* = 153)		Women ≥ 35 years (*N* = 82)		All men (*N* = 30)		All women (*N* = 235)		*P* values^*^
	*N*	%	*N*	%	*N*	%	*N*	%	*N*	%	*N*	%	
**Location**													
At workplace	*4*	*28.6*	*2*	*12.5*	46	30.7	31	36.5	6	20.0	77	32.8	0.21
At the university	*3*	*21.4*	*2*	*12.5*	9	6.0	9	10.6	5	16.7	18	7.7	0.16
At other school/training	*2*	*14.3*	*1*	*6.3*	12	8.0	6	7.1	*3*	*10.0*	18	7.7	0.72
Any workplace/university/school/training	6	42.9	*2*	*12.5*	51	34.0	38	44.7	8	26.7	89	37.9	0.31
In my home	*3*	*21.4*	*3*	*18.8*	17	11.3	16	18.8	6	20.0	33	14.0	0.41
In someone else’s home	*2*	*14.3*	*4*	*25.0*	19	12.7	7	8.2	6	20.0	26	11.1	0.23
In a public place, train etc.	8	57.1	4	25.0	98	65.3	27	31.8	12	40.0	125	53.2	0.18
Other place	*4*	*28.6*	*1*	*6.3*	24	16.0	14	16.5	5	16.7	38	16.2	1.0
**Offender**													
Regular partner	*2*	*14.3*	*4*	*25.0*	13	8.7	12	14.1	6	20.0	25	10.6	0.14
Temporary partner	*1*	*7.1*	*4*	*25.0*	19	12.7	4	4.7	5	16.7	23	9.8	0.34
Any partner	*3*	*21.4*	7	43.8	24	16.0	13	15.3	10	33.3	37	15.7	0.04
Family member or close relative	*1*	*7.1*	*3*	*18.8*	8	5.3	3	3.5	*4*	*13.3*	11	4.7	0.08
Person working at my workplace	*3*	*21.4*	*3*	*18.8*	32	21.3	15	17.6	6	20.0	47	20.0	1.0
Client, patient, customer pupil etc. at my workplace	*3*	*21.4*	*3*	*18.8*	22	14.7	21	24.7	6	20.0	43	18.3	0.81
Unknown person	8	57.1	6	37.5	103	68.7	30	35.3	14	46.7	133	56.6	0.33
Other person	5	35.7	*3*	*18.8*	22	14.7	17	20.0	8	26.7	39	16.6	0.20

* Chi square for difference between ‘all men’ and ‘all women’.

Note: Scania Public Health Cohort 2016. Values based on fewer than five observations are italicized.

The most commonly reported offender was an unknown person, 46.7% for men and 56.6% for women. A statistically significant gender difference was noted in that 33.3% of the men vs. 15.7% of the women reported having been exposed to SH by a partner. This difference was more pronounced in the older age group, with 43.8% (seven out of 16 exposed men) affirming this kind of exposure vs. 15.3% in the corresponding group of women. Furthermore, out of all exposed men (*N* = 30), four had been exposed by a family member or a close relative. This figure amounts to 13.3%, while this was true for only 4.7% of the women (*P* values for chi square difference 0.08).

Besides the mentioned observations, no clear age- or gender-related differences regarding location or offender were evident.

Table 3 presents the associations between, on the one hand, background factors and SH, and on the other hand, poor mental health, measured by three different assessment methods. Regarding the GHQ-case and SF-36 depression/anxiety case variables, about one out of four (GHQ) and one out of five (SF-36) persons in our sample scored above the level of ‘caseness’. A clear age gradient could be seen, with about three times higher risk in the youngest age group (18–24 years) compared with the oldest group (55 years and above), OR: 3.3 (95% CI: 2.7–4.0) and OR: 2.7 (95% CI: 2.2–3.4), for GHQ-case and SF-36 depression/anxiety case, respectively. As for self-labelled use of psychotropic medication, the age-related pattern differed. All in all, 16.8% of the participants had used such a drug during the past 3 months. The highest use, 18.2%, was reported in the oldest age group, which, according to their GHQ and SF-36 status, had the best mental health, and which therefore had been set as the reference group in this analysis. Those aged 25–54 years had statistically significant lower ORs for use, while the OR in the youngest group was OR: 0.8 (95% CI: 0.6–1.1).

**Table 3:** Background factors and exposure to sexual harassment during the past 12 months in relation to poor mental health, defined as (i) ‘GHQ-case’, (ii) ‘SF-36 depression/anxiety case’ and (iii) self-reported use of antidepressants, sedatives or hypnotics during the past 3 months

		GHQ-case (missing = 24)					SF-36 depression/anxiety case (missing = 443)					Use of psychotropic medication past 3 months				
		No	Yes	% Yes	OR	95% CI	No	Yes	% Yes	OR	95% CI	No	Yes or N/A	% Yes	OR	95% CI
Age groups	18–24	256	215	45.6	3.3	2.7–4.0	304	153	33.5	2.7	2.2–3.4	397	74	15.7	0.8	0.6–1.1
	25–34	479	391	44.9	3.2	2.7–3.7	599	258	30.1	2.3	2.0–2.8	748	122	14.0	0.7	0.6–0.9
	35–44	451	199	30.6	1.7	1.4–2.1	524	116	18.1	1.2	0.96–1.5	554	97	14.9	0.8	0.6–0.99
	45–54	800	349	30.4	1.7	1.5–2.0	895	231	20.5	1.4	1.2–1.6	975	175	15.2	0.8	0.7–0.97
	≥ 55	3659	936	20.4	1		3574	662	15.6	1		3779	838	18.2	1	
	Total	5645	2090	27.0			5896	1420	19.4			6453	1306	16.8		
Gender^a^	Men	2559	752	22.7	1		2659	481	15.3	1		2916	406	12.2	1	
	Women	3086	1338	30.2	1.4	1.3–1.6	3237	939	22.5	1.5	1.4–1.7	3537	900	20.3	1.9	1.7–2.1
Educational level^a^	≤ 12 y	2587	836	24.4	1		2546	652	20.4	1		2822	606	17.7	1	
	≥ 13 y	2918	1188	28.9	1.1	0.97–1.2	3252	729	18.3	0.8	0.7–0.9	3463	650	15.8	0.9	0.8–1.1
Born in Sweden^a^	Yes	5186	1830	26.1	1		5452	1216	18.2	1		5879	1148	16.3	1	
	No	421	233	35.6	1.5	1.3–1.8	415	187	31.1	2.0	1.6–2.4	514	143	21.8	1.5	1.2–1.8
Exposed to SH^a^	No	5415	1892	25.9	1		5668	1278	18.4	1		6126	1192	16.3	1	
	Yes	113	149	56.9	2.5	1.9–3.2	149	105	41.3	2.3	1.8–3.0	194	71	26.8	2.4	1.8–2.3

a ORs are age-adjusted.

Note: Scania Public Health Cohort 2016, *N* = 7759. Odds ratios (OR) and 95% confidence intervals (CI) for caseness and medication use are presented.

Female gender and being born outside Sweden were positively related to all aspects of poor mental health, while no association was seen between educational level and poor mental health. As for SH, the age-adjusted OR for being a GHQ-case was 2.5 (1.9–3.2), for the SF-36 measure of poor mental health, it was 2.3 (1.8–3.0) and for use of psychotropic medication 2.4 (1.8–2.3), in other words very similar size of the association between SH and mental health, regardless of how the latter was assessed. As a test of robustness regarding the association with SH, the mean scores of the continuous GHQ variable in 2016 were 1.46 and 3.60, respectively, for unexposed and exposed. The corresponding figures for GHQ in 2010 were 0.96 and 2.01. For the SF-36 variable, the scores were 75.20 and 61.17, respectively, for unexposed and exposed. In these *T*-tests, all differences were statistically significant with *P* values <0.0001.

Interaction analyses, performed with a dummy variable combining gender with SH exposure and using men not exposed to SH as the reference (OR = 1), showed that the unadjusted ORs for ‘GHQ-caseness’ were 1.8 (0.8–3.9) for men exposed to SH, for women not exposed to SH it was 1.4 (1.2–1.5) and for women exposed to SH it was 5.1 (3.9–6.7). This resulted in a synergy index of 3.4, thus indicating a positively modifying effect of female gender on the potential impact of SH on mental health. The same type of analysis was performed for the outcome ‘SF 36 depression/anxiety’, which yielded the synergy index of 4.5, and for the third outcome ‘Use of psychotropic drugs’, in which case the corresponding synergy index was 0.7. Thereafter synergy between exposure to SH and age (belonging to the age group ‘≤ 34 years’ vs. ‘≥ 35 years’) was tested in the same manner. In relation to the reference category (being ‘older’ and unexposed to SH), the OR for being older and exposed to SH was 2.5 (1.7–3.8); it was equally 2.5 (2.2–2.8) for being younger and unexposed to SH, and 6.3 (4.6–8.8) for being younger and exposed to SH. The resulting synergy index was 1.8. The corresponding synergy indexes regarding the other two outcomes were 1.2 for ‘SF 36 depression/anxiety’ and 1.1 for ‘Use of psychotropic drugs’.

In summary, being a woman seemed to be associated with a possibly greater vulnerability to SH exposure regarding poor mental health concerning two of the used outcome measures for poor mental health.

Table 4 presents a multivariate analysis, performed on those who had participated in surveys both in 2016 and in 2010, assessing the relationship between SH and poor mental health, measured as GHQ-case in 2016, with adjustment for age group, gender and, as a final step, ‘GHQ-case status’ 6 years earlier. The OR decreased, but was still statistically significant at 2.0 (1.4–3.0). The same type of analyses were performed with the ‘SF36 depression/anxiety case’ and ‘Use of psychotropic medication’ variables. The ORs in these cases were, unadjusted, 2.7 (1.8–4.1) and 1.9 (1.4–2.5), respectively, and after the final adjustment for status in 2010, 2.0 (CI 1.3–3.1) and 1.9 (1.4–2.6), respectively (data not shown in tables). Thus, the risk for developing poor mental health was doubled among those in our sample who reported exposure to SH during the past 12 months, regardless of which of the three types of assessment of the outcome that we used, and after adjustment for mental health (specifically for all three assessment methods) 6 years previously.

**Table 4:** Odds ratios (95% confidence intervals) for poor mental health, defined as ‘GHQ-case’, in 2016, in relation to exposure to sexual harassment during the past 12 months, with forward stepwise addition of age groups, gender, and GHQ-case status in 2010

		Model 1^a^		Model 2^b^		Model 3^c^		Model 4^d^	
		OR	95% CI	OR	95% CI	OR	95% CI	OR	95% CI
Exposed to sexual harassment	Yes vs. no	2.8	2.0–4.1	2.4	1.6–3.5	2.2	1.5–3.2	2.0	1.4–3.0
Age groups	≤ 34 years vs. ≥ 35 years			2.2	1.7–2.7	2.1	1.7–2.7	1.9	1.5–2.4
Gender	Women vs. men					1.4	1.2–1.5	1.3	1.1–1.4
GHQ-case in 2010	Yes vs. no							3.4	3.0–3.9

a Model 1: crude.

b Model 2: adjusted for age groups.

c Model 3: Model 2 + adjusted for gender.

d Model 4: Model 3 + adjusted for GHQ-case status in 2010.

**Note: Scania Public Health Cohort, *N* = 5886. OR, odds ratio;:** CI, confidence interval.

Finally, the population attributable fraction for poor mental health defined as GHQ-caseness was calculated to 13% for women 34 years and younger and to 0.6% for women 35 years and older.

## Discussion

This study showed that exposure to SH during the previous 12 months is associated with a higher risk for poor mental health, as measured by three different outcome methods. Furthermore, SH is particularly common for young women, among whom it may contribute to 13% of the caseload of the already high level of poor mental health. Moreover, it was shown that the most common setting for exposure to SH for both men and women are public places (including public transportation), but also that the workplaces and study environments constitute common areas. Thus, SH in Sweden is to a large extent manifested in the public arena, which strongly calls for further policy development and intervention efforts.

This study confirmed the observation that exposure to SH increases the risk for poor mental health.^8–12^ However, to the best of our knowledge, no previous study has demonstrated the robustness of this observation by using three different methods for defining poor mental health. The definition of SH presented to the respondents in this study was *‘Sexual harassment refers to all types of unwanted sexual advances directed towards oneself’*. This description conveys the notion of acts or verbal statements that are experienced as unwanted and therefore abusive or even threatening to the recipient. In other words, they can be described as an invasion of the individual’s integrity, which in general is regarded as a risk factor for poor mental health, especially if this invasion is grave or repeated. In this context, the finding of a greater ‘vulnerability’ to SH in women should be discussed. The same gender power structures that make SH a massively more common and serious issue for women may also render coping with SH a very disparate process for the genders. Thus, in one study, most men stated that being sexually ‘harassed’ at work was actually a positive experience, and that, if not, they could easily stop such a situation.^29^ This is in contrast to experiences of women exposed to SH, where fear, resentment and self-blame are common.^30^

The direction of causality might still be unclear. However, in a study following adolescents from 2010 to 2012, there was no clear support for the ‘reverse causation’ hypothesis.^10^ Other than studies focused on workplace organizational factors,^31^^,^^32^ there seems to be a scarcity of longitudinal general population studies assessing SH in relation to mental health. In our analysis with added data on baseline mental health status in 2010, we found that adjustment for poor mental health at baseline decreased the OR only marginally, or not at all. We therefore find it unlikely that reversed causation would explain our findings.

We computed the population attributable fraction for poor mental health among women in the age bracket 18–34 years, and arrived at an estimate of 13%. Thus, every eighth case would be caused by SH if our estimate is reasonably accurate. This is a noteworthy level, considering that the prevalence of poor mental health is higher among young women than in any other broadly defined section of the general population. It makes the experience of SH one of the most important contributors to poor mental health among young women. Considering that the studied exposure most likely has a cumulative effect (i.e. the impact on mental health is very likely to last into the future), the calculated magnitude of the contribution to poor mental health might be underestimated.

We also found that SH exposure occurred in public spaces, as well as in workplaces and educational institutions; i.e. it is a pervasive phenomenon, which makes it difficult to actively avoid on the individual level. This, together with the marked association with subsequent poor mental health, makes SH an important issue for gender policy as well as for public health policy and intervention and certainly underlines the importance of using the #MeToo momentum as a window of opportunity. According to the policy theorist Kingdon,^33^ when the stream of politics (e.g. the #MeToo movement), the stream of problems (evidence mapping and analyzing the phenomenon) and the stream of policy (interest of policy makers to deal with the issue) intersect, there is a high likelihood that the issue will enter the policy agenda.

## Strengths and limitations of the study

The main strengths of this study are that it is based on a fairly large general population sample comprising both men and women, that it used validated instruments, that the outcome was measured by three different methods, that reasonable control for confounding could be made, that it could yield a comprehensive picture of SH in terms of location of occurrence and type of perpetrator and, finally, that the possibility of reverse causation could be tested in a subpopulation of the analyzed sample. Regarding the weaknesses of our study, we particularly want to clarify that we did not use concrete examples when assessing the prevalence of SH. It is well-known that inquiries detailing descriptions of potentially offensive behaviours will yield higher prevalence rates than self-labelling of SH with one single item. This phenomenon has been explained as the result of a general unwillingness to admit victimization^34^ or to the normalization of harassment behaviour in the work environment.^35^^,^^36^

Given the strong contextual influence of what is considered SH,^30^^,^^37^ there seems to be a general consensus in the research community that there is no ‘gold standard’ definition of SH.^38^ Nevertheless, most instruments are likely to be able to distinguish between groups that are ‘more’ exposed and groups that are ‘less’ exposed, which allow us to determine patterns of distribution and effects on health of SH.

When we stratified the data regarding location of the SH event and type of offender, and split this information for age and sex (table 2), the numbers were very small in some categories. We regard those analyses as interesting for an overall assessment of group differences. However, this information was not used for addressing the main objective of this study.

Missing information is a potential source of selection bias. However, in this study, such missing information is generally very low (0.3–5.7% for single variables, and 9.3% for all variables in the fully adjusted multivariable model), and thus, not indicating a potentially important source of bias in our analyses.

Other limitations are the level of non-response, although there is no apparent reason to assume considerable bias regarding the studied exposures and outcomes, and the essentially cross-sectional study design, although the mentioned check for reverse causation could be made by available longitudinal information. Using data from the 2010 data collection for baseline information on caseness could invite bias due to differential drop-out between the cohort waves, although a separate study showed that this effect was marginal.

## Conclusions

The results of this study make SH an important issue for gender policy as well as for public health policy and intervention.

## Funding

This work was funded by FORTE (Swedish Research Council for Health, Working Life and Welfare; Grant Number 2015-00885) and the Medical Faculty at Lund University.

*Conflicts of interest*: None declared.

Key points

- The association between exposure to sexual harassment and poor mental health was verified by three different well-validated outcome measures.
- Women were both much more frequently exposed to sexual harassment than men, and in addition more vulnerable regarding the development of poor mental health after such an exposure.
- Exposure to sexual harassment may contribute to 13% of the burden of poor mental health among Swedish women 18–34 years of age.
- The results of this study make SH an important issue for gender policy as well as for public health policy and intervention.

## References

[1] Latcheva R. Sexual harassment in the European Union: a pervasive but still hidden form of gender-based violence. J Interpers Violence 2017;32:1821–52. 30156991 10.1177/0886260517698948

[2] Quick JC , McFadyen MA. Sexual harassment: have we made any progress? J Occup Health Psychol 2017;22:286–98. 27732009 10.1037/ocp0000054

[3] Bondestam F , Lundqvist M. Sexual harassment in higher education – a systematic review. Eur J High Educ 2020;10:397–419.

[4] Gekoski A , Gray JM, Adler JR, Horvath MAH. The prevalence and nature of sexual harassment and assault against women and girls on public transport: an international review. J Criminol Res Policy Pract 2017;3:3–16.

[5] DelGreco M , Ebesu Hubbard AS, Denes A. Communicating by Catcalling: power Dynamics and Communicative Motivations in Street Harassment. Violence Against Women 2021;27:1402–26. 32567540 10.1177/1077801220927085

[6] Clear ER , Coker AL, Cook-Craig PG, et al Sexual harassment victimization and perpetration among high school students. Violence Against Women 2014;20:1203–19. 25288593 10.1177/1077801214551287

[7] Ranganathan M , Wamoyi J, Pearson I, Stöckl H. Measurement and prevalence of sexual harassment in low- and middle-income countries: a systematic review and meta-analysis. BMJ Open 2021;11:e047473. 10.1136/bmjopen-2020-047473 PMC8231049 34168030

[8] Bucchianeri MM , Eisenberg ME, Wall MM, et al Multiple types of harassment: associations with emotional well-being and unhealthy behaviors in adolescents. J Adolesc Health 2014;54:724–9. 24411820 10.1016/j.jadohealth.2013.10.205 PMC4107652

[9] Landstedt E , Gillander GK. Experiences of violence among adolescents: gender patterns in types, perpetrators and associated psychological distress. Int J Public Health 2011;56:419–27. 21544531 10.1007/s00038-011-0258-4

[10] Dahlqvist HZ , Landstedt E, Young R, Gådin KG. Dimensions of peer sexual harassment victimization and depressive symptoms in adolescence: a longitudinal cross-lagged study in a Swedish sample. J Youth Adolesc 2016;45:858–73. 26910524 10.1007/s10964-016-0446-x PMC4826406

[11] Magnusson Hanson LL , Nyberg A, Mittendorfer-Rutz E, et al Work related sexual harassment and risk of suicide and suicide attempts: prospective cohort study. BMJ 2020;370:m2984. 32878868 10.1136/bmj.m2984 PMC7463167

[12] Rugulies R , Sorensen K, Aldrich PT, et al Onset of workplace sexual harassment and subsequent depressive symptoms and incident depressive disorder in the Danish workforce. J Affect Disord 2020;277:21–9. 32781365 10.1016/j.jad.2020.06.058

[13] Nielsen MB , Einarsen S. Prospective relationships between workplace sexual harassment and psychological distress. Occup Med (Lond) 2012;62:226–8. 22394681 10.1093/occmed/kqs010

[14] Muhonen T. Exploring gender harassment among university teachers and researchers. J Appl Res High Educ 2016;8:131–42.

[15] Hogh A , Conway PM, Clausen T, et al Unwanted sexual attention at work and long-term sickness absence: a follow-up register-based study. BMC Public Health 2016;16:678. 27475538 10.1186/s12889-016-3336-y PMC4967501

[16] Sexuell och reproduktiv hälsa och rättigheter (SRHR) i Sverige 2017. Resultat från befolkningsundersökningen 2017. (Sexual and reproductive health and rights in Sweden 2017. Results from the population-based survey SRHR2017) Folkhälsomyndigheten (Public Health Agency of Sweden); 2019. Available at: https://www.folkhalsomyndigheten.se/publicerat-material/publikationsarkiv/s/sexuell-och-reproduktiv-halsa-och-rattigheter-i-sverige-2017/?pub=60999 (11 November 2021, date last accessed).

[17] 65 metoo-grupper lämnar kravlista till regeringen (65 metoo groups deliver list of demands to the government): Sveriges Television (a public service television channel) 2018. Available at: https://www.svt.se/nyheter/inrikes/65-metoo-grupper-lamnar-kravlista-till-regeringen (11 November 2021, date last accessed).

[18] Carlson L. #MeToo in Sweden – over 75,000 voices raised. SSRN (Social Science Research Network). 2021. doi:10.2139/ssrn.3802572

[19] Hansson K , Sveningsson M, Ganetz H. “We passed the trust on”: strategies for security in #MeToo activism in Sweden. European Society for Socially Embedded Technologies (EUSSET); 2019. doi:10.18420/ecscw2019_ep14

[20] Carlsson F , Merlo J, Lindström M, et al Representativity of a postal public health questionnaire survey in Sweden, with special reference to ethnic differences in participation. Scand J Public Health 2006;34:132–9. 16581705 10.1080/14034940510032284

[21] Canivet C , Nilsson A, Björk J, et al Assessment of selection bias due to dropouts in the follow-up of the Scania Public Health Cohort. Scand J Public Health 2021;49:457–64. 32466718 10.1177/1403494820919544 PMC8135243

[22] Nilsson A , Bonander C, Strömberg U, et al Reweighting a Swedish health questionnaire survey using extensive population register and self-reported data for assessing and improving the validity of longitudinal associations. PLoS One 2021;16:e0253969. 10.1371/journal.pone.0253969. 34197538 PMC8248630

[23] Goldberg DP , Gater R, Sartorius N, et al The validity of two versions of the GHQ in the WHO study of mental illness in general health care. Psychol Med 1997;27:191–7. 9122299 10.1017/s0033291796004242

[24] Goldberg DP , Oldehinkel T, Ormel J. Why GHQ threshold varies from one place to another. Psychol Med 1998;28:915–21. 9723146 10.1017/s0033291798006874

[25] Ware JE Jr , Sherbourne CD. The MOS 36-item short-form health survey (SF-36). I. Conceptual framework and item selection. Med Care 1992;30:473–83. 1593914

[26] Matcham F , Norton S, Steer S, Hotopf M. Usefulness of the SF-36 Health Survey in screening for depressive and anxiety disorders in rheumatoid arthritis. BMC Musculoskelet Disord 2016;17:224. 27215696 10.1186/s12891-016-1083-y PMC4878044

[27] Rothman K. Modern Epidemiology. Boston, MA: Little, Brown & Co, 1986.

[28] Rockhill B , Newman B, Weinberg C. Use and misuse of population attributable fractions. Am J Public Health 1998;88:15–9. 9584027 10.2105/ajph.88.1.15 PMC1508384

[29] Berdahl JL , Magley VJ, Waldo CR. The sexual harassment of men? Exploring the concept with theory and data. Psychol Women Quart 1996;20:527–47.

[30] Gutek BA. Understanding sexual harassment at work. Notre Dame J Ethics Public Policy 1992;6:335–58.

[31] Topa Cantisano G , Dominguez JRM, Depolo M. Perceived sexual harassment at work: meta-analysis and structural model of antecedents and consequences. Span J Psychol 2008;11:207–18. 18630662 10.1017/s113874160000425x

[32] Willness CR , Steel P, Lee K. A meta-analysis of the antecedents and consequences of workplace sexual harassment. Pers Psychol 2007;60:127–62.

[33] Rawat P , Morris JC. Kingdon’s “Streams” model at thirty: still relevant in the 21st century? Politics and Policy 2016;44:608–38.

[34] Nielsen M , Bjørkelo B, Notelaers G, Einarsen S. Sexual harassment: prevalence, outcomes, and gender differences assessed by three different estimation methods. J Aggress Maltreat Trauma 2010;19:252–74.

[35] Sojo VE , Wood RE, Genat AE. Harmful workplace experiences and women’s occupational well-being: a meta-analysis. Psychol Women Quart 2016;40:10–40.

[36] Folke O , Rickne J, Tanaka S, Tateishi Y. Sexual harassment of women leaders. Daedalus 2020;149:180–97.

[37] Timmerman G , Bajema C. Sexual harassment in northwest Europe – a cross-cultural comparison. Eur J Womens Stud 1999;6:419–39.

[38] McDonald P. Workplace sexual harassment 30 years on: a review of the literature. Int J Manag Rev 2012;14:1–17.

